# A Unique Case of Frontotemporal Dermoid Cyst Presenting as Orbital Cellulitis

**DOI:** 10.7759/cureus.37050

**Published:** 2023-04-03

**Authors:** Joseph P Menousek, Tyler Pistone, Arnett Klugh, III, James Vargo, Judith Wong

**Affiliations:** 1 Neurosurgery, University of Nebraska Medical Center, Omaha, USA; 2 Plastic and Reconstructive Surgery, University of Nebraska Medical Center, Omaha, USA; 3 Neurosurgery, Miller Children's & Women's Hospital, Long Beach, USA

**Keywords:** digeorge syndrome, orbital cellulitis, dermoid cyst, frontotemporal, pediatric

## Abstract

Dermoid cysts are benign developmental anomalies that can occur anywhere along the neuroaxis or embryonic lines of fusion. While intracranial dermoid cysts at the midline frequently have an associated nasal or subcutaneous sinus tract, it is quite rare to encounter an intracranial dermoid cyst off the midline with a lateral sinus tract. Standard practice for the treatment of dermoid cysts is surgical resection to minimize the risks of meningitis, abscess, mass effect, neurologic deficit, and/or death.

A 3-year-old male with a history of DiGeorge syndrome presented with right orbital cellulitis and a right-sided dermal pit. Computed Tomography (CT) imaging demonstrated a dermal sinus tract with an associated lytic bone lesion within the right sphenoid wing and posterolateral orbital wall with intracranial extension. The patient was taken to the operating room in conjunction with plastic surgery for resection of the dermal sinus tract and intraosseous dermoid.

This case presents a rare occurrence of a non-midline, frontotemporal dermal sinus tract associated with a dermoid cyst with intracranial extension presenting with pre- and post-septal orbital cellulitis. Important considerations include preservation of the frontal branch of the facial nerve, preservation of orbital structure and volume, complete surgical resection to prevent infectious complications including meningitis, and a multidisciplinary surgical approach with plastic surgery, ophthalmology, and/or otolaryngology.

## Introduction

Dermoid cysts are benign developmental anomalies originating embryologically from the entrapment of ectodermal elements consisting of a stratified squamous epithelium lining containing keratin and hair [1]. Intracranial dermoid cysts are classically found in the midline [2], though a few rare case reports have demonstrated non-midline dermoid cysts [3-5]. It is crucial to identify and surgically resect these cysts associated with dermal sinus tracts as there exists the possibility for infection and meningitis. In this case report, we outline a unique case of a frontotemporal dermal sinus tract associated with an intracranial dermoid cyst presenting as orbital cellulitis in a patient with DiGeorge Syndrome. 

## Case presentation

History

**Figure 1:**
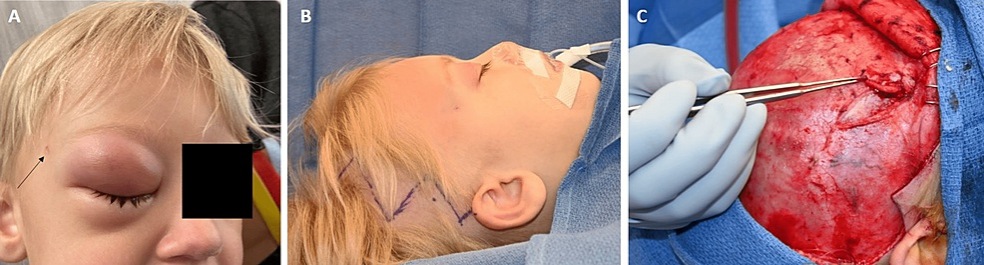
Clinical photographs of the patient with right-sided orbital cellulitis. The dermal sinus tract is denoted by the black arrow (A). Intraoperative photograph of planned incision (B). Demonstration of the dermal sinus tract extending into the bony canal (C).

The patient presented as a 3-year-old male with a past medical history including DiGeorge syndrome, single cardiac ventricle anatomy following aortic reconstruction, Glenn, and Damus-Kaye-Stansel procedures, heart block with pacemaker, T-cell lymphopenia, and gastrostomy tube dependence, in addition to developmental delay and intellectual disability. He presented to the emergency department with a fever and 24 hours of progressively worsening swelling and erythema of the right eye in addition to mild proptosis concerning for orbital cellulitis. Of note, pupils were equal in size and reactive to light and the patient was without obvious visual field deficit. Interestingly, the parents reported a history of a skin dimple over the right pterion that has been present since birth that intermittently extruded hair and purulent material (Figure 1).

The patient was febrile on presentation (38.3°C) with associated leukocytosis (14.65 thousand). Other pertinent laboratory data included C-reactive protein (CRP) of 5.0 mg/dL (normal 0.0 - 0.7 mg/dL), erythrocyte sedimentation rate (ESR) of 18 mm/h (normal 1-15 mm/h), and neutrophil count 10.66 thousand (normal 1.80-7.40 thousand). Blood cultures obtained in the emergency department demonstrated no growth, though antibiotics were started prior to the acquisition of blood cultures. The patient was also started on intravenous steroids in addition to intravenous vancomycin and ceftriaxone for broad-spectrum antibiotic coverage for presumed orbital cellulitis. 

Preoperative evaluation

**Figure 2:**
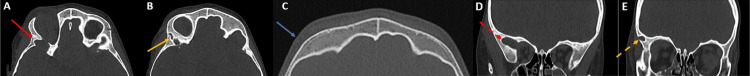
Pre-operative axial and coronal CT without contrast The dermoid cyst is seen within the sphenoid bone denoted by the solid red arrow (A) and dotted red arrow (D) with bony erosion posteriorly into the temporal pole and medially into the orbit. The dermal sinus tract is faintly visible within the soft tissues of the scalp superior and lateral to the sphenoid wing (C). Its associated dermal sinus tract is seen coursing through the sphenoid bone denoted by the solid yellow arrow (B) and dotted yellow arrow (E).

Given his history of cardiac pacemaker placement, computed tomography (CT) imaging of the head and orbits with and without contrast was obtained as he was not a candidate for magnetic resonance imaging (MRI). The obtained images demonstrated an intraorbital, extraconal rim-enhancing fluid collection along the superolateral wall of the right orbit measuring approximately 1.4 x 1.4 x 0.5cm immediately adjacent to a lytic bone lesion within the right sphenoid wing/posterolateral orbital wall with intracranial epidural extension anterior to the right temporal pole. This was associated with a circuitous cutaneous tract extending through the right temporal soft tissue (Figure 2).

Considering the complex presentation, a multidisciplinary team was assembled to review the case and develop a surgical plan including neurosurgery, plastic surgery, ophthalmology, infectious disease, otolaryngology, and critical care medicine. Given the concern for an infected intracranial/intraorbital dermoid cyst and orbital abscess, the decision was made to proceed with a right craniotomy for resection of the dermal sinus tract and sphenoid mass. 

Operative treatment

The patient was taken to the operating room in conjunction with pediatric plastic and reconstructive surgery. The frontal branch of the facial nerve was percutaneously mapped based on surface landmarks and found its course in close proximity to the deeper portion of the tract. Considering this, preoperative discussion with parents included the risk of transient neuropraxia versus permanent frontal branch palsy. An incision was made circumferentially around the sinus tract and blunt dissection with limited cautery was used to dissect along the tract while minimizing the risk of injury to the facial nerve. Dissection was carried through the subcutaneous tissue and temporalis muscle down to the periosteum. At this point, a coronal incision was made (Figure 1) and the scalp flap was raised using the interfascial technique for frontalis branch preservation [6]. The temporalis muscle was elevated in a sub-periosteal plane from its origin and was reflected inferiorly. At this point, the tract was delivered through the coronal flap and temporalis muscle and was visualized entering a bony canal (Figure 1). A standard pterional craniotomy was then performed. The sinus tract was initially located within the craniotomy and drilled out to preserve the tract in continuity, however, it was ultimately divided due to the significant circuity of its course. Ultimately, the cystic, inflamed, pink-tan-colored mass was identified in the lateral sphenoid bone with intracranial extension into the middle fossa with adhesions to the dura. Erosion of the posterolateral orbital wall was also observed. A lateral orbitotomy was then performed to gain access to the right orbit and purulent material was drained from the sub-periosteal space and was sent for culture. There was no violation of the orbital periosteum and no evidence of extraconal infection. After complete visualization of the mass, it was dissected from the temporal dura using standard microsurgical technique. There was no obvious intradural extension. The resultant bony defect was then reconstructed with autologous split calvarial bone grafts, the temporalis muscle was re-suspended, and the scalp was closed in anatomic layers. 

Postoperative course

The patient was monitored in the intensive care unit. Post-operative CT imaging demonstrated resection of the mass and anatomic cranial and orbital reconstruction. The patient did have transient neuropraxia of the right frontalis muscle following the operation. Intraoperative cultures revealed gram-positive cocci on gram stain though no growth was observed. Subsequent broad-range PCR demonstrated positivity for *Porphyromonas endodontalis*. Infectious disease initiated a six-week course of intravenous ceftriaxone, daptomycin, and oral metronidazole. He recovered appropriately and was discharged home on postoperative day seven in stable condition. 

Pathologic analysis

**Figure 3:**
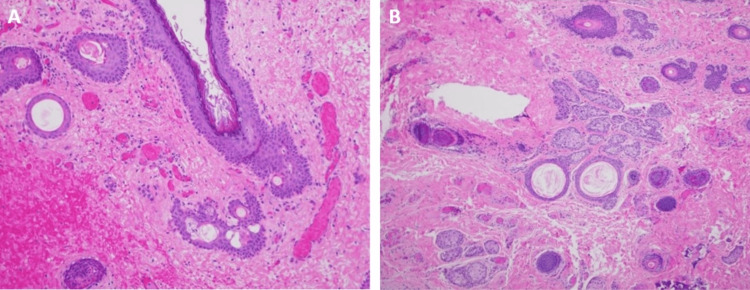
Pathologic analysis of intraoperative specimens Dermal sinus tract with a portion of skin and underlying tissue with a squamous epithelium lined tract (A). The dermoid cyst with ruptured and inflamed structure with focal presence of squamous epithelium and keratinous debris with associated foreign body type giant cell reaction (B).

Pathologic analysis of the tract and mass revealed “portion of skin and underlying tissue with a squamous epithelium lined tract” and “ruptured and inflamed structure with focal presence of squamous epithelium and keratinous debris with associated foreign body type giant cell reaction” (Figure 3). 

## Discussion

Dermoid cysts are benign inclusion cysts that arise from ectodermal tissue and may be associated with a dermal sinus tract [1]. Dermal sinus tracts typically occur in the midline at the rostral or caudal end of the developing neural tube as they are formed due to failure of separation of cutaneous ectoderm from neuro-ectoderm at the time of neural tube closure. Dermoid cysts can involve both the bone of the skull and the dura mater. If these masses involve the bone, they typically form within the diploic space and grow at a linear rate expanding both the inner and outer table [7]. Dermoid cysts comprise 15.4%-58.5% of all pediatric skull tumors [8], and most are diagnosed in children less than five years of age [8]. We are aware of a few case reports of patients with frontotemporal dermoid cysts discovered after the development of orbital cellulitis [3, 5, 8-13]. However, there are no reports of such an occurrence in a patient with DiGeorge syndrome. 

The presentation of dermoid cysts varies from the discovery of a benign cutaneous pit with extrusion of keratinous debris to local or systemic infection. These masses can also rupture and, depending on the location, lead to chemical meningitis secondary to the inflammatory nature of fat and keratin. Patients may also exhibit cerebrospinal fluid leakage from the sinus tract, and there have been reports of cranial nerve palsy caused by sequelae of dermoid cysts and associated sinus tracts [10]. As the sinus tracts can sometimes be inconspicuous, diagnosis may be difficult to make in the face of much larger or more obvious symptoms as was the case in the patient described in this report. 

The presence of dermoid cysts can be confirmed with skull radiographs, CT, and MRI. Dermoid cysts are typically osteolytic with well-defined, sclerotic margins on skull radiographs. CT imaging demonstrates hypodense lesions since the cysts typically contain fat. On MRI, dermoid cysts are hypointense on T1-weighted imaging and T2-weighted imaging and can exhibit marked diffusion restriction. These lesions typically do not contrast enhance. Dermoid cysts may also be associated with developmental anomalies such as Klippel-Feil anomaly, encephaloceles, coccygeal sinus, and venous sinus anomalies [10]. Given the presence of a pacemaker in this patient, MRI was unable to be obtained, though his CT imaging studies were consistent with the above (Figure 2). 

The treatment of choice for dermoid cysts is surgical resection [1]. A multidisciplinary team-based surgical approach involving plastic and reconstructive surgery, ophthalmology, and/or otolaryngology is recommended in some cases, especially when the facial structures, nose, orbit, or temporal bone is involved. Care must be taken to avoid rupture of the dermoid cyst, especially when intracranial, given the risk of chemical meningitis. The entire mass and associated dermal sinus tract, if present, must be removed in their entirety to minimize the risk of recurrence. There are no documented cases of these masses extending into the subdural space, though a few reports have described adhesion to adjacent dura as was present in the patient described in this report [5]. 

Another important consideration during the resection of frontotemporal dermoid cysts is the preservation of the frontal branch of the facial nerve. Before making an incision, the frontal branch can be mapped using cutaneous landmarks or using compound muscle action potentials [14]. Further protection of this nerve can be accomplished during exposure using an interfascial dissection when raising the scalp and muscle from the skull. While temporary neuropraxia may still occur secondary to traction or surgical manipulation, permanent frontal branch palsy is rare [15]. 

Pathogens associated with dermal sinus tract infections have been documented in prior case reports. These include *Staphylococcus aureus* [16], *Actinomyces*, *Peptoniphilus*, *Propionibacterium* species [17], and *Escherichia coli* [10]. There has been one prior report of a patient with sacrococcygeal dermal sinus tract associated with polymicrobial meningitis where the causative agent was *Porphyromonas* species, in addition to *Finegoldia*, *Campylobacter*, and *Bacteroides* [18]. The genus of *Porphyromonas* in our patient was of a different species, however. Infectious complications include posterior fossa abscess, ventriculitis, meningitis, osteomyelitis, orbital cellulitis, and epidural abscess [19] as seen in this patient.

## Conclusions

In this report, we document a rare occurrence of a non-midline, frontotemporal dermal sinus tract associated with a dermoid cyst with intracranial extension presenting with pre- and post-septal orbital cellulitis. These lesions commonly go unnoticed until severe symptoms arise as was the case in this patient. Prompt recognition and surgical resection of these masses are the sole means of treatment, thereby minimizing the risk of potentially life-threatening intracranial and orbital infections. 
